# Observation and rating HEMS Crew in Non-Technical Skills, CRM Medical Simulation in Norwegian Air Ambulance

**DOI:** 10.1186/1757-7241-23-S2-A21

**Published:** 2015-09-11

**Authors:** Jan Martinsen

**Affiliations:** 1 Norwegian Air Ambulance Foundation, Drøbak, Norway

## Introduction

“Camp Torpomoen” is an annual training camp for HEMS crew in Norwegian Air Ambulance. The main goal is to improve medical treatment of the sick and injured patient. To obtain this, the crew practice in Medical Simulation, team training in a patient scenario, creating realistic environments.

The objective for this study was to observe and rate the non-technical skills of the crew in a medical trauma scenario. The results give an implication on the status of the crew performance on CRM.

## Method

The method is a behavioral marker system developed by psychologists and anesthetists through a research project in Scotland. The system rates skills in non-technical elements for anesthetists in teams, for example interaction and lead. Used integrally with medical knowledge and clinical skills, non-technical skills will help to support safe and efficient treatment both in everyday tasks and in critical situations.

For further information of the system and rating, please follow this link http://www.abdn.ac.uk/iprc/ants/

The training scenario had two learning goals: a clinical (technical) goal and a social/cognitive (non-technical) goal. This observational study only comprises the latter.

Individual skills and performance were included in the overall assessment of the crew. A 4-point scale was used to describe the level of performance demonstrated, 4 being the best. Four categories were observed and given scores: Task management, Team work, Situation awareness and Decision-making. In addition, 15 elements under these categories wererated.

For rating criteria, see the link above. Each crew consisted of an anesthetist as the team leader, a HCM, and a pilot, which is the normal crew concept in the air ambulance service.

## Results

In total, 28 crews were observed and rated.

The results showed that the non-technical skills for the 28 crews ranged between 3.37 and 3.57, with **an average value of 3.51**. Rating for the 15 elements varied between 2.0 and 4.0 for all crews.

## Conclusion

The results show that the skills are based on a combination of knowledge in CRM and understanding their role as a team-member.

Rates in the range of 3+ indicate that the performance was of an **acceptable** standard but could be improved.

